# Branched Polymeric Prenucleation Assemblies Initiate Calcium Phosphate Precipitation

**DOI:** 10.1021/jacs.4c07325

**Published:** 2024-09-04

**Authors:** Ertan Turhan, Ieva Goldberga, Christopher Pötzl, Waldemar Keil, Jean-Michel Guigner, Martin F.T. Haßler, Herwig Peterlik, Thierry Azaïs, Dennis Kurzbach

**Affiliations:** † Institute of Biological Chemistry, Faculty of Chemistry, University of Vienna, Währinger Str. 38, Vienna 1090, Austria; ‡ Vienna Doctoral School in Chemistry (DoSChem), University of Vienna, Währinger Str. 42, Vienna 1090, Austria; § CNRS, Laboratoire de Chimie de la Matière Condensée de Paris (LCMCP), Sorbonne Université, 4, Place Jussieu, Paris F-75005, France; ∥ Institut de Minéralogie et Physique des Milieux Condensés (IMPMC), Sorbonne Université, 4, Place Jussieu, Paris F-75005, France; ⊥ Faculty of Physics, University of Vienna, Boltzmanngasse 5, Vienna 1090, Austria; # Vienna Doctoral School in Physics (VDS), University of Vienna, Boltzmanngasse 5, Vienna 1090, Austria

## Abstract

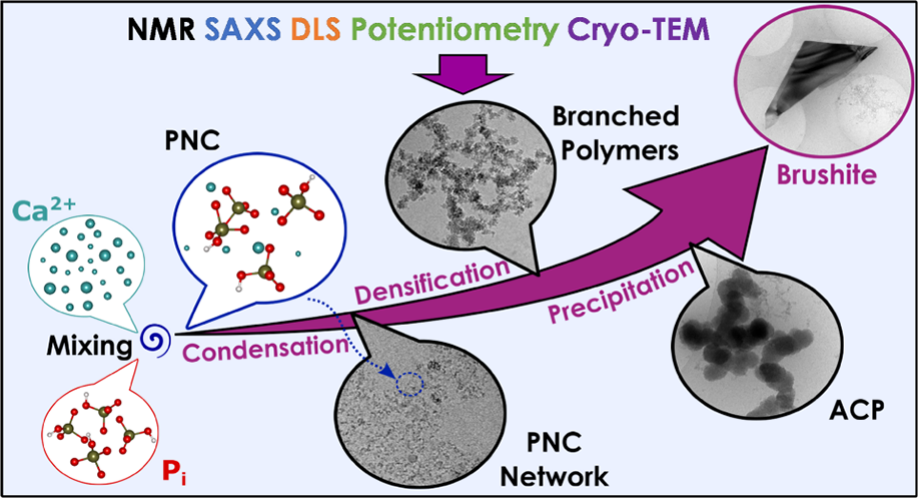

The formation of crystalline calcium phosphate (CaP) has recently gained ample attention as it does not follow the classic nucleation-and-growth mechanism of solid formation. Instead, the precipitation mechanisms can involve numerous intermediates, including soluble prenucleation species. However, structural features, stability, and transformation of such solution-state precursors remain largely undisclosed. Herein, we report a detailed and comprehensive characterization of the sequential events involved in calcium phosphate crystallization starting from the very early prenucleation stage. We integrated an extensive set of time-resolved methods, including NMR, turbidimetry, SAXS, cryo-TEM, and calcium-potentiometry to show that CaP nucleation is initiated by the transformation of “branched” polymeric prenucleation assemblies into amorphous calcium phosphate spheres. Such a mineralization process starts with the spontaneous formation of so-called nanometric prenucleation clusters (PNCs) that later assemble into those branched polymeric assemblies without calcium ion uptake from the solution. Importantly, the branched macromolecular species are invisible to many techniques (NMR, turbidity, calcium-potentiometry) but can readily be evidenced by time-resolved SAXS. We find that these polymeric assemblies constitute the origin of amorphous calcium phosphate (ACP) precipitation through an unexpected process: spontaneous dissolution is followed by local densification of 100–200 nm wide domains leading to ACP spheres of similar size. Finally, we demonstrate that the timing of the successive events involved in the CaP mineralization pathway can be kinetically controlled by the Ca^2+^/P_i_ molar ratio, such that the lifetime of the soluble transient species can be increased up to hours when decreasing it.

## Introduction

The classical nucleation-and-growth paradigm describing the formation of ionic solids has recently been challenged by the observation of (meta)stable solution-state precursors, known as prenucleation clusters (PNC).^1−3^ These dynamic and nanosized ionic solution-state self-assemblies were observed to precede the formation of solid materials. However, despite their critical role, the transformation processes undergone by PNC leading to solid materials are still poorly understood, although crucial for apprehending PNC reactivity. These processes are particularly debated for crystalline solids that adopt amorphous phases as transient precursors upon nucleation via a PNC pathway, e.g., calcium carbonates^4−7^ or calcium phosphates.^8−14^

In addition, since their discovery, PNC have been observed at the onset of the formation process of a wide array of inorganic or hybrid materials from silica,^15^ metal–organic frameworks (MOFs),^16^ zeolites,^17^ to cyclosilicates,^18^ and sulfates.^19^ It is speculated that PNC can act as early-stage templates in material formation processes.^20^ In other words, their structural solution-state properties would be retained upon material formation and predetermine, to some degree, the structure and function of the final solids. This intriguing property enables the conceptualization of rational materials design strategies based on the manipulation of mineral precursors,^20^ warranting the clarification of the reaction processes involving PNC at the molecular level.

This task has yet to be tackled as PNC are highly dynamic entities with often poorly defined structures, making them experimentally very challenging to characterize. In particular, the observation of various condensed solution-state phases complicates their depiction. Prenucleation entities may be described as ion pairs,^21^ ionic clusters (Posner clusters^22,23^ or calcium triphosphate^8,14^ in the case of apatite formation), polymeric self-assemblies,^8,13,14,22−26^ or at the origin (and eventually as constituents) of distinct condensed liquid phases.^27−30^

As a result, the definition of the notion “non-classical” crystallization and the characterization of PNS is currently an important topic. The sizes of different transient ionic clusters, the kinetics of transformation, and the pathways to the final solid are all debated, in particular, whether these observed phenomena agree with classical nucleation-and-growth theories or not (see, e.g., refs^31−34^). Herein, we yet avoid making speculations about the theoretical definition of the observed phenomena and whether they are considered classic or nonclassic and focus on the interpretation of the available data.

We investigate the time-dependent structural dynamics of prenucleation species involved in calcium phosphate (CaP) formation, employing a suite of time-resolved methods, integrating *in situ*^31^P nuclear magnetic resonance (NMR), turbidimetry, real-time small-angle X-ray scattering (SAXS), dynamic light scattering (DLS), calcium-potentiometry, and cryogenic transmission electron microscopy (cryo-TEM). The combination of NMR, calcium-potentiometry, and advanced TEM techniques has already shown a strong potential for revealing the presence, kinetic behaviors, and quantities of PNC in biomineralization contexts.^14,30,35^ In addition to the techniques mentioned above, we demonstrate the prowess of real-time SAXS for comprehending the transitions of PNC into nucleated solids. Thus, we reveal, under generic experimental conditions, the existence of transient species with high ion density that are the key players in the nucleation process of calcium phosphate. The highlighted species can be visually described as “branched” polymeric assemblies chronologically occurring between individual PNC and amorphous calcium phosphate (ACP). Several features of the observed phase transition are notable. First, the transition from individual PNC to “branched” polymeric assemblies proceeds without Ca^2+^ uptake, suggesting a PNC aggregation mechanism. Second, the resulting dense solute phase remains stable for up to 5 h before material solidification occurs. Finally, the transition from “branched” polymeric assemblies to ACP spheres proceeds through a dissolution-reprecipitation mechanism, as evidenced by cryo-TEM observations.

The latter point is particularly remarkable as the ubiquitous concept of amorphous solid nucleation emerging through simple aggregation of PNC (with or without additional ion uptake)^2^ is not observed under our conditions and may need to be updated with alternative solidification pathways.

## Results

In the following, we will first describe the kinetics of the successive events involved in CaP formation and then characterize the time-dependent structure of the condensed transient phases. It should be noted that the exact definition (and distinction from other phenomena, such as liquid–liquid phase separation or ionic self-assembly) of nonclassical nucleation and prenucleation clusters is still debated. In the following, we use the notion of PNC in line with earlier research in the field conducted under similar conditions^9,10,13,14,24,35^ to generically describe a metastable ionic species that forms prior to precipitation of solid CaP. However, classifications into theories or regimes of saturation are avoided.

## *In Situ* Monitoring of Successive Mineralization Events during CaP Precipitation

**Figure 1:**
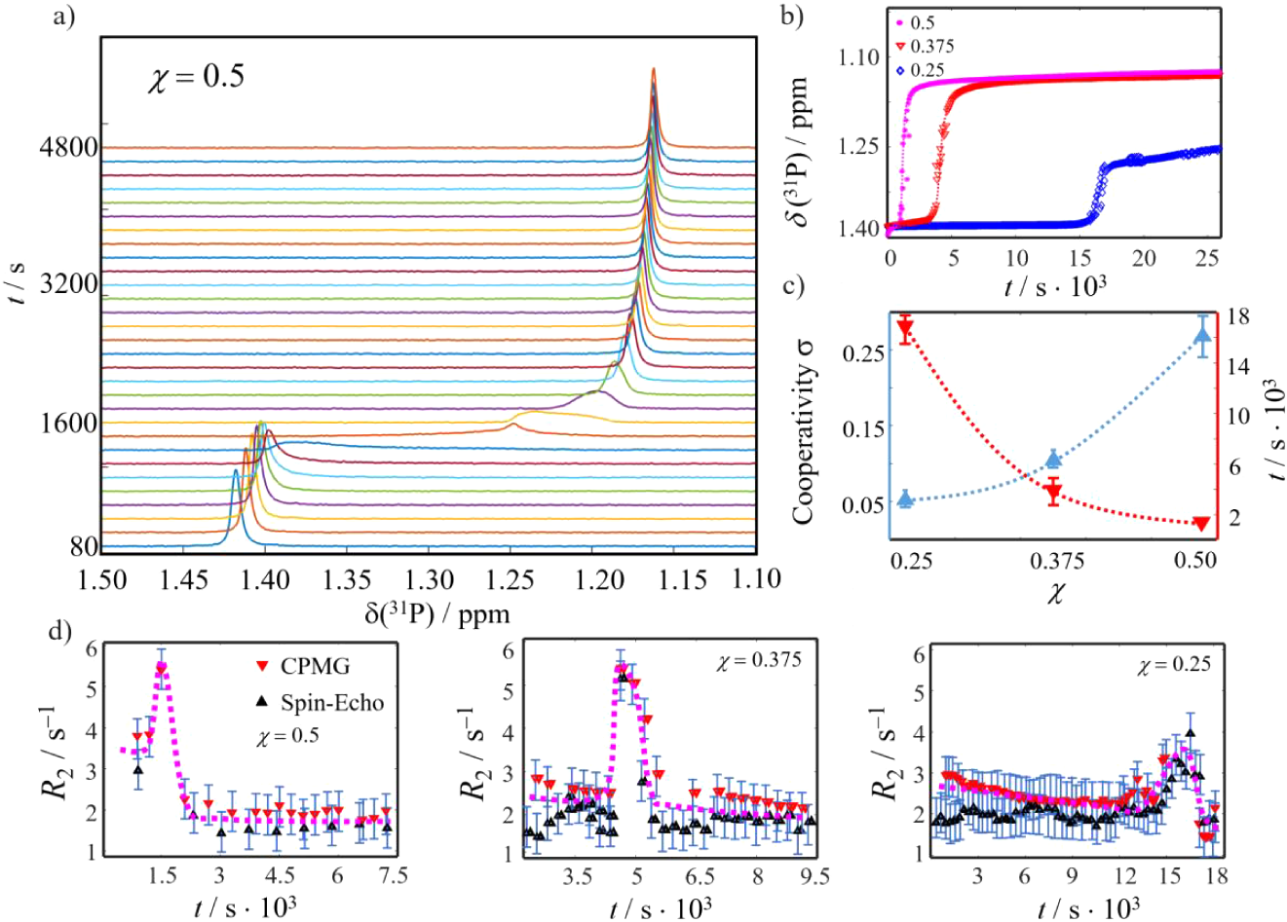
a) Exemplary real-time ^31^P spectra for χ = 0.5. b) ^31^P chemical shifts as a function of time for χ = 0.5, 0.375, and 0.25. The dashed lines indicate the sigmoidal fits. c) Transition times (red) and the cooperativity parameter (σ; blue) for the three different molar ratios obtained from the fits shown in panel (b). d) Transverse relaxation rate constants *R*_2_ for the three probed molar ratios (recorded with a spin echo (red) or a CPMG pulse sequence (black) to suppress chemical exchange effects). During the NMR-observable transition, the relaxation rates increase in both cases, indicating that chemical exchange does not underlie the observed effects.

Real-time ^31^P NMR spectroscopy has already shown potential for CaP prenucleation species monitoring in earlier reports.^14^ Herein, we use this method to probe three different mineralizing solutions with constant inorganic phosphate (P_i_) concentrations ([P_i_] = 8 mM). Only calcium ion concentrations were varied ([Ca^2+^] = 2, 3, and 4 mM), leading to Ca^2+^/P_i_ mole fractions of χ = 0.25, 0.375, and 0.5, respectively. We observed that varying ion concentrations led to different time delays between the preparation of the samples and the onset of NMR-detectible CaP formation, even with >4 h differences for the lowest mole fraction χ. Figure 1a shows a representative time series of ^31^P NMR spectra for χ = 0.5 at pH 7 and 25 °C in 20 mM HEPES-buffered solution (all other data can be found in Figure SI1 of the Supporting Information). After its preparation, the sample was immediately inserted into the NMR spectrometer. Detection started ca. 3 min after the insertion (corresponding to *t* = 0 in Figure 1a,b). Then, a ^31^P spectrum was recorded every 80 s (averaging 16 FID per spectrum using a recovery delay of 5 s). The ^31^P signals experience (*i*) a time-dependent upfield chemical shift change, indicating a rearrangement of the local coordination sphere around the P_i_ ions, (*ii*) a loss in NMR signal intensities, and (*iii*) an intermediate line broadening accompanying the chemical shift change. With decreasing molar ratio χ (Figure 1b,c), these transitions occur later and slower in agreement with previous observations showing that CaP nucleation kinetics by PNC in solution can be described by a simple collision model directly linked to P_i_ and Ca^2+^ concentrations.^11^ Here, the onset of the transitions occurred at ca. 1200, 2500, and 15 000 s after sample preparation for χ = 0.5, 0.375, and 0.25, respectively. Remarkably, the time-dependent change in chemical shift resembled a cooperative phase transition (Figure 1b,c) with a typical sigmoidal kinetic behavior. Fitting the time dependence of the chemical shift to a sigmoidal function (see the Experimental section), higher cooperativity values (σ) were obtained for larger molar ratios (Figure 1c). σ describes the pace of the phase transition event, in other words, the slope in Figure 1c. Hence, a higher χ is correlated with higher cooperativity and, thus, faster transition events.

The sudden transitions in the chemical shift are also concomitant with a transient increase of *R*_2_ transverse relaxation rate constants (Figure 1d), indicating the formation of larger CaP intermediates that restrict the rotational freedom of the P_i_ units and, thus, broaden lines (see Figure SI2 for the line widths) before the CaP aggregates become too large for solution-state detection.^35^ Interestingly, the *R*_2_ rate constants are also higher before the transition than after it. As will be shown further, this observation can be accounted for by P_i_ being bound in small PNC before the transition, while only residual free P_i_ remains in solution after the end of the transition event.

**Figure 2:**
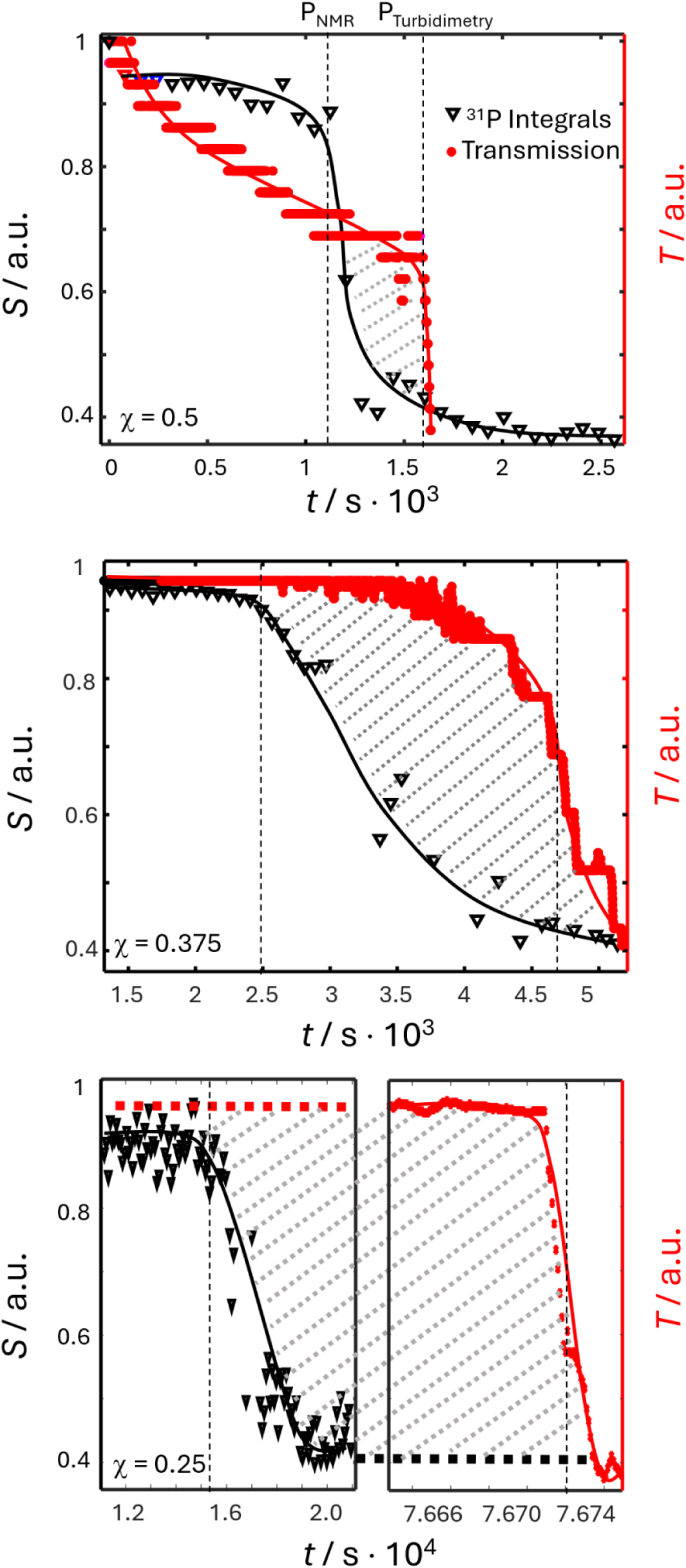
Normalized ^31^P signal integrals *S* (black) together with light transmission intensities *T* (red) obtained from turbidimetry for χ = 0.5, 0.375, and 0.25.

To understand how the NMR-detected transitions correlate with the formation of solid CaP particles, we employed real-time turbidimetry measurements, sensitive to particles with diameters >450 nm (Figure 2). Interestingly, these experiments revealed that large solid CaP particles form only with a significant delay to the sigmoidal signal loss observed by NMR (cf. Figure 1). For the three probed conditions, the time differences were found to be 400, 2100, and 61 700 s, respectively. The gray dashed lines in Figure 2 indicate the periods between the NMR and turbidimetry-detected transitions. During these periods, self-assembled CaP condensates remain suspended with sizes beyond the NMR sensitivity limit^14^ yet too small for detection by turbidity measurements. In other words, CaP can form a persistent condensed phase in aqueous systems, much like DOLLOP arrangements predicted for calcium carbonates or soluble arrangements observed for calcium carbonates in the presence of amino acids.^30,36^

The combined NMR and turbidimetry data point toward several distinct time periods. Immediately after sample preparation, the NMR and the turbidimetry data do not show any strong variations. The system is composed of solute species. Then, the sigmoidal transition of the NMR time traces highlights the formation of an NMR-invisible condensed CaP state. After this transition step, the NMR and the turbidimetry data again do not change. Finally, the sample turbidity increases due to a second transition, i.e., the formation of solid CaP particles of size > 450 nm.

With increasing mole fraction χ, each time interval shortens, and the transition between them shifts toward shorter times (Table 1). This indicates an acceleration of kinetics for each mineralization step, i.e., both induction events (NMR and turbidimetry data are stable) and transition events (NMR or turbidimetry data change) shift toward shorter periods/time points.

**Table 1:** Approximate Transition Times and Durations during CaP Mineralization Observed by Real-Time SAXS, Turbidimetry and ^31^P NMR^a^

χ	*p*_NMR_	*p*_Turbidimetry_	*P*_*1*_ (SAXS)	*P*_*2*_ (SAXS)	*P*_*3*_ (SAXS)	*P*_*3*_ (DLS)	*d*_2_ (SAXS)	*d*_3_ (SAXS)
0.5	1200	1600	1100	1800	2200	2200	700	400
0.375	2500	4600	1400	2000	4000	4000	600	2000
0.25	15 000	76 700	13 500	15 000	17 500	14 000^b^	1500	2500

a All values are reported in seconds. Note that *P*_1_ = *d*_1_.

b Note that the signal was very noisy under these conditions and that this value needs to be evaluated with caution.

## The Transition from Solute Prenucleation Phases to Solid Particles

To better understand the observed phase transition processes, we employed *real-time* SAXS. This method is ideally suited to bridge the gap between the NMR and turbidimetry length scales (ca. 2–100 nm) and provides information about the organization of the CaP condensates. Recent work on CaCO_3_ formation has already demonstrated the prowess of SAXS in unraveling the dehydration of PNC and their evolution into the first amorphous calcium carbonate phase.^37^

For SAXS, the samples were prepared the same way as for the NMR experiments, filled into sealable glass capillaries, and then immediately transferred to the SAXS equipment. 2D SAXS images were recorded for 120 s for χ= 0.5 and 0.375, and for 900 s for χ= 0.25 to take the differential kinetics into account. Data were radially integrated and background corrected to result in SAXS intensities, which could be fitted to a power law function *I*(*q*) = *a· q*^–n^ with amplitude *a* and fractal dimension *n*. To visualize the development of scattering intensities, original data for χ= 0.5 are presented in Figure SI3, and an error analysis is presented in Figure SI4.

**Figure 3:**
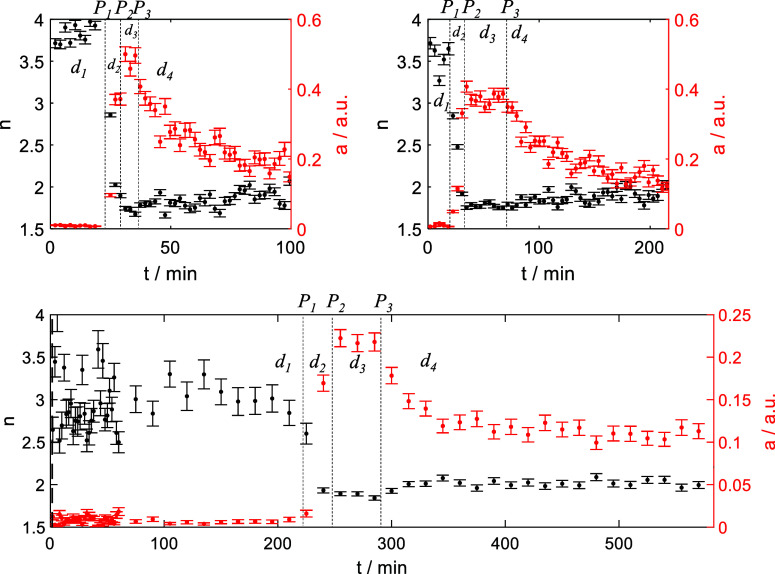
Fractal dimension *n* (black) and amplitude *a* (red) from a power law fit of SAXS scattering intensities for χ = 0.5, 0.375, and 0.25 in the *q*-range between *q* = 0.1 and 1 nm^–1^. The profiles could also be dissected into four periods, *d*_1_ to *d*_4_, separated by time points *P*_1_ to *P*_3_ (see Table 1). For a more detailed error assessment of fit errors, see the Supporting Information.

Figure 3 shows the time-dependence of the fit parameters *a* (red symbols, right scale) and *n* (black symbols, left scale) for the different mole fractions χ. Four characteristic periods (labeled *d*_1_ to *d*_4_) are discernible, separated by time points *P*_1_ to *P*_3_ (see Figure SI5 for a comparison of the timing time delays found by NMR, turbidimetry, and SAXS experiments).

During *d*_1_, the fractal dimensions remain stable at 3.5 < *n* < 4 in all three cases, corresponding to objects exceeding the length scale of SAXS (about 100 nm) with a smooth surface (*n* = 4), a fractal surface or an object with additional density fluctuations (3 < *n* < 4),^38,39^ which can also correlate with a fluctuation in electron densities.^40^ It should be noted that the fractal dimension of 4 in SAXS experiments has recently been correlated with a liquid–liquid phase separation (LLPS)-type event.^41^ It is, thus, possible that the herein observed initial transition can be classified as LLPS.

Further, note that differences in fractal dimensions are often correlated with the occurrence of DLCC (diffusion-limited cluster–cluster growth) or RLCC (reaction-limited cluster–cluster growth) polymer growth models. However, the difference between DLCC and RLCC in terms of fractal dimension is often very small.^42^ The herein-presented SAXS data do not allow for such a distinction to be made due to the time-resolved manner of data acquisition and the accompanying limitations in signal-to-noise ratios (SNR).

During time interval *d*_2_, the fractal dimension changes to *n* ≈ 2. This can be interpreted as a phase transition leading to a two-dimensional morphology, such as a polymeric structure or a branched condensate.^37^ This change is accompanied by a sudden increase in amplitude *a*. The endpoint of this interval *P*_2_ corresponds to the beginning of the transition observed by NMR (Figure 1b).

Then, during *d*_3_, no changes in the two-dimensional structures were observed. The amplitude and fractal dimension remained constant. This interval corresponds roughly to the time between the NMR-observed and turbidimetry-observed transitions (Figure 2) except for the χ = 0.25 condition.

Finally, during *d*_4_, an exponential decrease of the SAXS intensity is observed (fractal dimension stays at *n* ≈ 2) concomitant to the changes in turbidity (Figure 2). This phenomenon is caused by a second phase transition, i.e., the formation of large solid CaP particles starting from the solute polymeric phase existing during *d*_3_. An exception is observed for the χ = 0.25 condition. The two transitions (seen by turbidimetry and SAXS) are significantly shifted. This is most likely due to the slowed-down kinetics for χ = 0.25 that lead, at the beginning of *d*_4_, to the nucleation of small particles of size <450 nm invisible to turbidimetry until they reach the critical size >450 nm after ∼76 000s.

The sequence of events is rather similar for each probed mole fraction χ but with a considerable increase in the *d*_1_–*d*_4_ duration with decreasing χ. Table 1 lists the characteristic times and compares these to the NMR and turbidimetry experiments. Given the precision of our multimodal experiments, all timings match as expected.

It should be noted that heterogeneous surfaces (such as those of the used glass capillaries) can influence solid formation by inducing nucleation and generating artifacts.^37^ However, given that we performed the experiments with different devices, which house very different sample volumes (ranging from 5 μL to 20 mL), and that the timings of the observed transitions were coherent between the different experiments, surface effects are unlikely to be a dominant bias.

**Figure 4:**
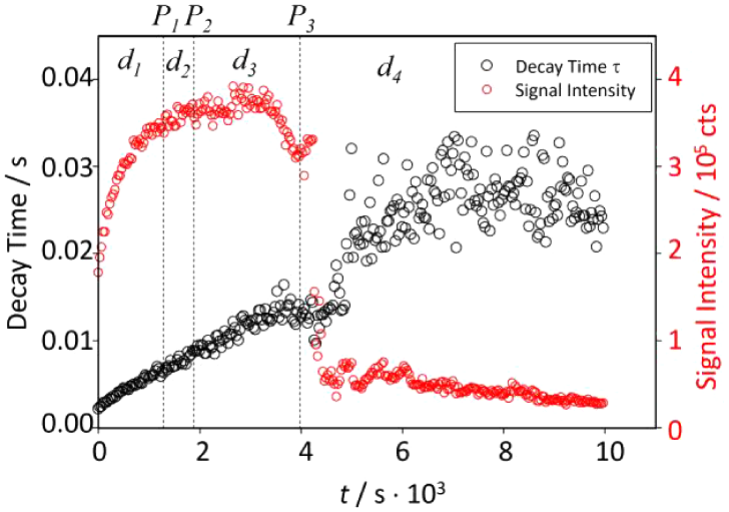
Dynamic light scattering intensities and decay times for χ= 0.375. The signal intensities (red curve) gradually increase before exhibiting a fast drop after ca. 4000 s (coinciding with turbidity and SAXS measurements). The decay time (black), proportional to the product of fluid viscosity and particle size, increases linearly, then reaches a plateau before becoming unstable (due to low signal intensity) after the transition point at 4000 s (see Table 1 for the other proved conditions).

Dynamic light scattering (DLS) experiments, which are sensitive to larger particles beyond the SAXS length scale, confirmed the growth of CaP particles during *d*_1_*–d*_3_. Figure 4 shows time traces of decay times and signal intensities for χ = 0.375. Most importantly, a steady increase of the signal intensity is observed (detector at 90°) during *d*_1_, which underlines our above interpretation of the presence of particles during this phase exceeding the SAXS length scale. The increasing intensity indicates that the amount of these early-stage precursors continuously grows. Then, during *d*_3_, the intensity remained constant before suddenly decreasing at the beginning of *d*_4_. This observation again points toward a phase transition in which large solid CaP particles are formed.

**Figure 5:**
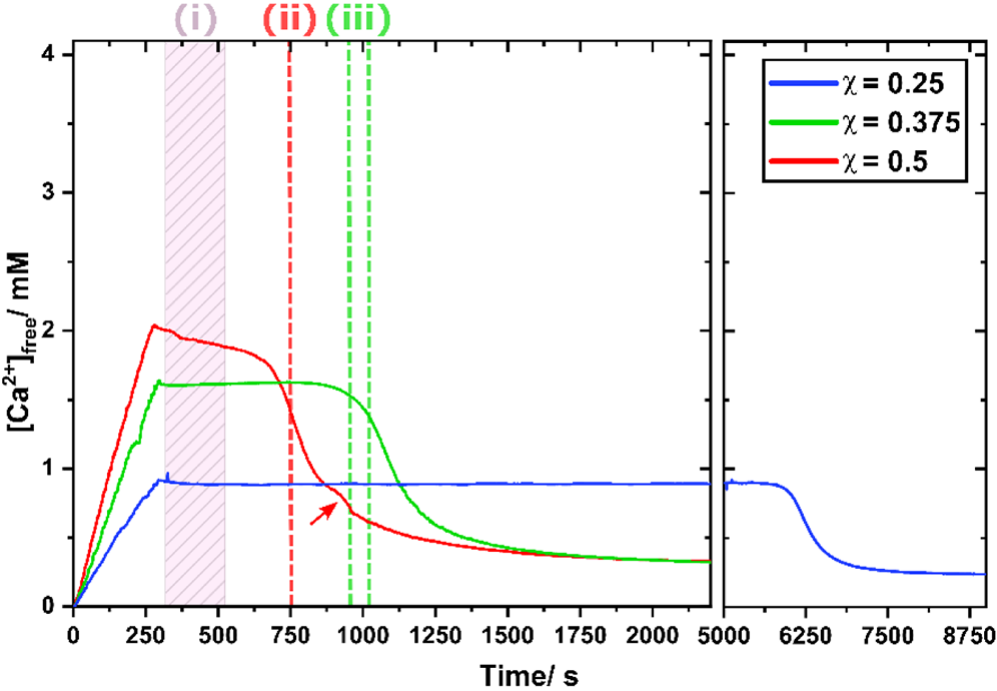
Ca-potentiometry measurement for the three probed mineralizing conditions. Dashed lines indicate theoretical free Ca^2+^ concentration in the absence of soluble prenucleation species. Vertical lines ((i), (ii), and (iii)) indicate sampling time points for cryo-TEM (see main text). For an estimate of the free Ca^2+^ concentration in the absence of any phosphate, see Figure SI7.

In the next step, calcium potentiometry was used to monitor the Ca^2+^ concentrations during CaP mineralization to supplement the scattering data from the viewpoint of the Ca^2+^ counterions. First attempts were made without stirring, but the calcium-potential measurements were unstable during the investigated time frame. This led to unreliable results (Figure SI6), likely resulting from insufficient homogeneity around the Ca^2+^-selective electrode. Hence, calcium-potentiometry monitoring was carried out under slow stirring (120 rpm) after the controlled addition of a Ca^2+^ solution into phosphate solution stirred at 5 mL/s (all other details can be found in the Experimental section). The resulting data are shown in Figure 5.

For all three probed conditions, qualitatively similar profiles were observed, each with two major features. (*i*) A plateau in free calcium ion concentration after complete mixing of the solutions and (*ii*) a rapid drop in [Ca^2+^] concomitant with sample precipitation. In agreement with the NMR and scattering results, the reaction kinetics accelerated with the Ca^2+^/P_i_ ratio. Interestingly, the free [Ca^2+^] measured during the plateau phase were systematically lower than the expected value. Only 50, 57, and 53% of the total [Ca^2+^] remained free in solution for χ = 0.5, 0.375, and 0.25, respectively (Figure SI7). This behavior is attributed to the formation of CaP prenucleation clusters in solution. However, we note that the PNC-bound Ca^2+^ concentrations measured under our conditions (∼50% of the total calcium in solution is bounded into prenucleation species) are much higher than in previous studies where values varied between 5 and 15%.^8,11^ Clearly, though, the Ca-potentiometry-derived mineralization kinetics indicate (*i*) the existence of soluble prenucleation species in equilibrium with free Ca^2+^ during the plateau phase followed by (*ii*) a Ca^2+^ uptake during the precipitation event.

**Figure 6:**
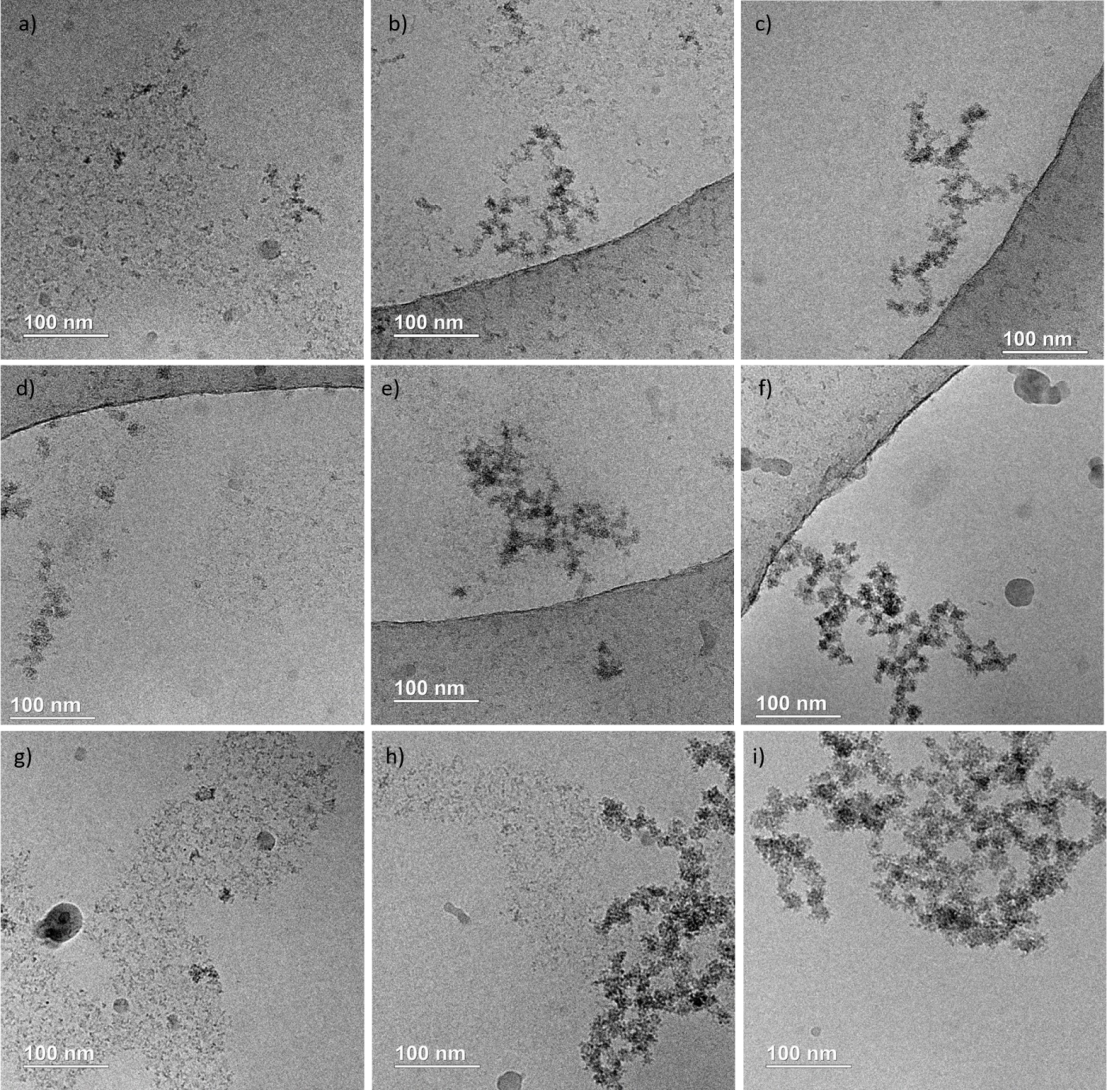
CryoTEM observations at the onset of the mineralization reaction displaying individual PNC (a, d, g), coexistence of PNC and polymeric branched aggregates (b, e, h), and polymeric branched aggregates (c, f, i) for the following conditions: (a–c) χ= 0.25, (d–f) χ = 0.375 and (g–i) χ = 0.5. Solutions were cryofixed at 20 s (χ = 0.5), 60 s (χ = 0.375), and 120 s (χ = 0.25) after complete mixing.

To learn more about the size and morphology of the different species involved in the mineralization events, we carried out cryo-TEM observations at specific time points chosen according to calcium-potentiometry curves (see Figure 5). First, we analyzed the solutions a few minutes after mixing. We found PNC with a size of ∼3 nm for each condition (Figure 6a,d,g). These PNC frequently assemble into an extended network of hundreds of nm (see also Figure SI8**)**. In addition, we also observed the coexistence of individual PNC with supramolecular structures in the form of branched polymeric assemblies of 100–300 nm in size resulting from the aggregation of individual clusters (Figures 6b,e,h; c,f,i; SI9 and 10).

The Ca-potentiometry data suggest that these polymeric assemblies form spontaneously from PNC without Ca^2+^ uptake. These observations agree with previous reports of similar polymeric assemblies, which were observed during hydroxyapatite formation both in simulated body fluid and in oversaturated conditions where a reaction-limited aggregation of individual prenucleation complexes was suggested.^8,13^ Importantly, individual PNC and branched polymeric assemblies are also observed under non-stirring conditions before the precipitation (Figure SI10), which shows that slow stirring only impacts the reaction rate but not the morphologies of successive species.

We want to highlight that comparable polymeric suprastructures have also been evidenced in the context of CaCO_3_ formation. In particular, it has been predicted, using computer simulations combined with the analysis of extensive experimental data, that large assemblies can form spontaneously in solution. These species were described as ionic polymers (so-called DOLLOP for dynamically ordered liquid-like oxyanion polymer), composed of alternating calcium and carbonate ions, with a dynamic topology consisting of chains, branches, or rings.^26^ It was further shown that some specific phosphate-based molecular additives can promote the stabilization of PNC and their association with calcium ions while at the same time allowing the further binding of carbonates.^43^ Likewise, *in situ* TEM observations have revealed that l-aspartic acid stabilizes prenucleation clusters of ∼2 nm in size as well as dynamic suprastructures of about 10–20 nm, constituted by PNC, for at least 15 min before precipitation.^30^

**Figure 7:**
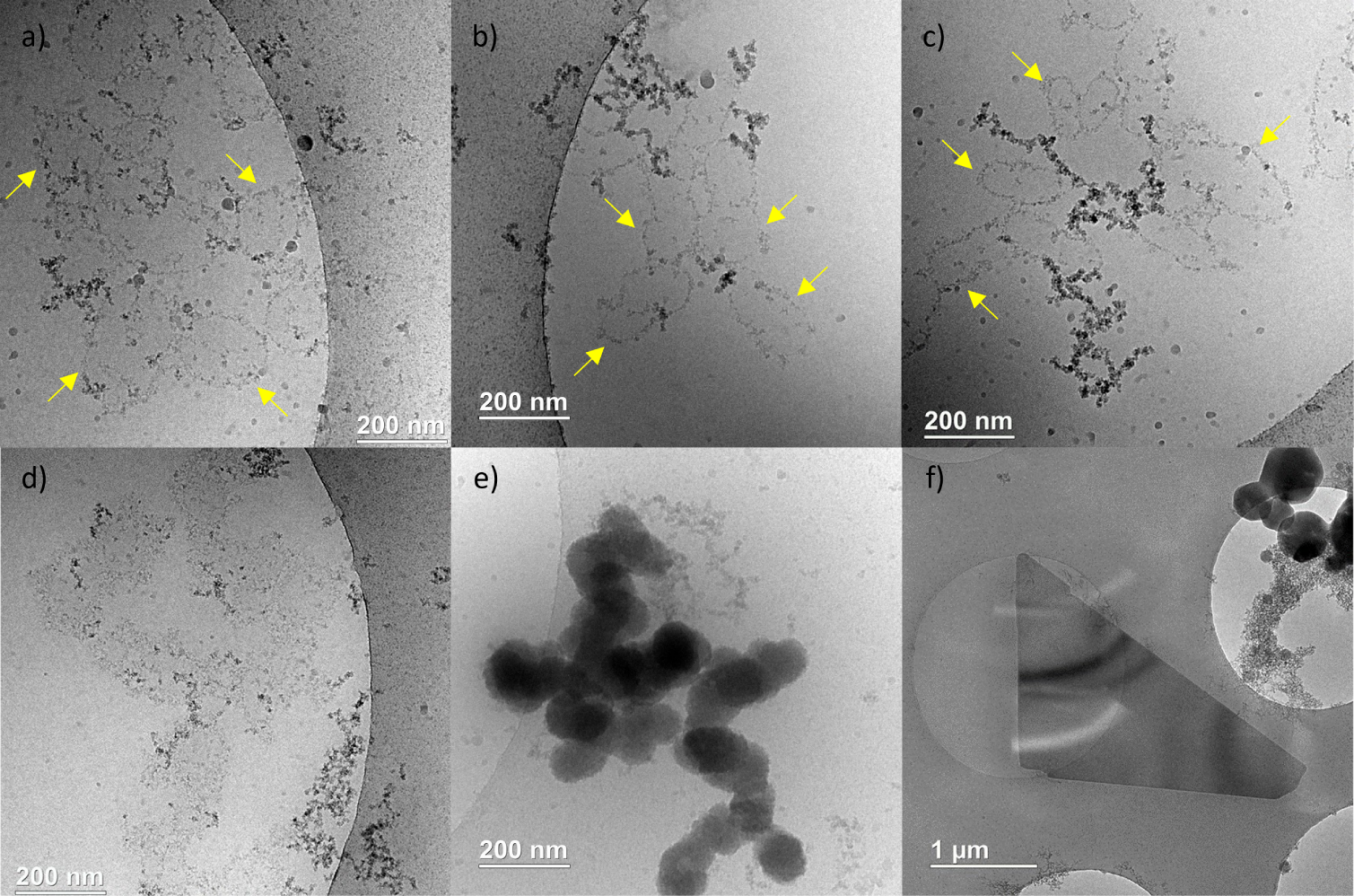
Cryo-TEM observations at the beginning (a–e) and at the end (f) of the precipitation event displaying: circular domains (yellow arrows) for condition (a) χ = 0.375 (600 s of reaction), (b, c) χ = 0.5 (450 s of reaction), (d) denser circular domains (χ = 0.375, 840 s of reaction), (e) aggregation of ACP-like spheres (χ = 0.5, 450 s of reaction), and (f) brushite platelets (χ = 0.5, 2 h).

To assess the transition from the initial species to nucleated phases, further observations were made during the precipitation events, 450 s (χ = 0.5), 600 s (χ = 0.375), and 840 s (χ = 0.25) after completion of the mixing step (cf. Figure 5). Interestingly, we still observed branched polymeric aggregates but together with circular domains delimited by a more contrasted zone (yellow arrows in Figure 7a–c). The diameters of these structures are ranging between 100 and 200 nm. Note that the circular domains tend to increase in contrast and to densify in structures of similar size (Figure 7d). Finally, we observe aggregates made of dense ACP-like spheres still of similar diameters ranging between 100 and 200 nm (Figure 7e).

These observations help to clarify the nucleation process, starting with the PNC. Here, we want to highlight the crucial step corresponding to the densification of circular/spherical domains leading to ACP spheres, as the first nucleated phase. The Cryo-TEM observations point to a “dissolution-reprecipitation” process. However, in the specific case of a dissolution step of the polymeric assemblies, an increase of the free Ca-concentration should coincide, which is yet not observed in the potentiometry experiments. However, if the dissolution step is very fast and concomitant with the reprecipitation step, the Ca-activity increase can be masked by the subsequent Ca^2+^ uptake. Hence, ACP spheres formation could involve a local reorganization of the ions into the branched polymeric assemblies, concomitant with a Ca^2+^ uptake and possibly coupled to a local dehydration step, leading to local densification of circular/spherical domains, which gain density until forming ACP. Strikingly, our observations disagree with the often-suggested nucleation via direct aggregation of PNC,^8,13^ at least under our conditions.

Lastly, cryo-TEM showed that the ACP spheres transform into large micrometric crystalline plates (Figures 7f and SI11) corresponding to brushite crystals (CaHPO_4_·2H_2_O), as confirmed by EDX and XRD analyses. The ACP-to-brushite transition also proceeds through a dissolution-reprecipitation process as a characteristic slope discontinuity is observed in the calcium-titration curve.

## Discussion

Combining the results from all the above-mentioned techniques, CaP mineralization comprises four distinct periods of dynamic evolution and two distinct phase transitions:

*d*_1_: During this period (starting immediately after sample preparation), NMR and turbidimetry data remain largely constant. SAXS indicates large inhomogeneities with a surface from smooth (*n* = 4) for the highest (χ = 0.5) to rough (*n* = 3.5) for the lowest (χ= 0.25) concentration ratio. At the same time, DLS shows an increasing decay time and intensity due to the growth of larger inhomogeneities. Cryo-TEM observations agree with this interpretation, showing the existence of ∼3 nm large PNC (∼50% of the total amount of Ca^2+^) that can gather into large “cloudy” assemblies of hundreds of nm. The separation process leads to a dense phase (with a higher calcium phosphate content) and a less dense phase, which is visible as clouds in the TEM images in Figure 6a,d,g. In contrast, the dehydrated nanoparticles appear as polymeric branched aggregates in Figure 6c,f,i. However, it should be noted that the density of the aggregates, as well as their fractal nature, cannot be unambiguously derived from these 2D images, and a quantitative correlation with the scattering pattern in the SAXS experiments cannot be made without speculation. Further, a mass fractal-type morphology would lead to form factor-like features in the SAXS profiles, which have not been observed.^40^ Hence, the fractal dimension of >3 possibly stems from heterogeneous electron density due to the clustering of PNC into cloud-like formations.

The presence of smaller PNC (coexisting with the larger aggregates) is further supported by *in situ*^31^P *R*_2_ experiments, which show an increased relaxation rate during *d*_1_ compared to free phosphate in solution (Figure 1).

*d*_2_: SAXS shows a fast phase transition from *n* = 4 to 2, indicating the formation of polymeric two-dimensional structures developing from the assembly of PNC. These structures were then observed by cryo-TEM, revealing branched polymeric assemblies (∼100–200 nm) made of individual clusters. The transition from PNC to polymeric assemblies thereby proceeded without Ca^2+^ uptake (evidenced by calcium potentiometry). The transition is also reflected in the sudden increase in the ^31^P *R*_2_ rate constant upon particle growth before the CaP condensates become too large for detection by solution-state NMR. Interestingly, comparable observations have already been made in the context of CaSO_4_ solidification,^44,45^ in particular initially forming large-scale domains, and their further collapse was observed.

*d*_3_: NMR chemical shifts, sample turbidity, and SAXS amplitudes and dimensions remained constant during this delay, corresponding to the period over which the branched polymeric assemblies stay stable.

*d*_4_: DLS intensity decreases suddenly due to a second phase transition, i.e., the formation of solid CaP particles, and the evaluation of the decay time is unstable due to this phase transition. In SAXS, an exponential decay of the intensities is observed, from which a typical time constant for the aggregation time is derived. Concomitantly, the sample turbidimetry increases, confirming the formation of particles of size >450 nm, except for the χ = 0.25 condition. For the latter, the transition is significantly shifted due to the formation of smaller solid particles invisible to turbidimetry until they reach the critical size for detection. Cryo-TEM identifies the first solid particles as ACP sphere aggregates that evolve into brushite platelets. The transition from polymeric assemblies to solid ACP spheres is identified by cryo-TEM as a dissolution-reprecipitation process. The concomitant Ca^2+^ uptake identified by calcium potentiometry can be the origin of the local increase in ionic density (circular/spherical domains of 100–200 nm in size) observed by cryo-TEM just after the local dissolution of the branched aggregates.

Variations of the Ca^2+^/P_i_ ratio do not influence the event cascade in terms of the morphologies of the transient species or phase transition sequences. The same transient species leads to the formation of brushite under all probed conditions. However, the kinetics are strongly affected, showing an acceleration with an increase in χ. Interestingly, the soluble transient species observed under our conditions were already observed during hydroxyapatite formation by Habraken,^8^ pointing toward common characteristics in the formation process of both calcium phosphate polymorphs, brushite, and hydroxyapatite. Interestingly, the initial pH values are close to neutral in the two studies (7.4 in ref^8^ vs 7.0 in our study), which can explain the similar initial behaviors. However, the final pH values after completion of CaP formation that impact the final crystalline phase, were slightly acidic pH (pH = 6.8–6.9) in our study explaining brushite formation, in contrast to hydroxyapatite formation observed at a final pH > 7 in ref.^8^. To the best of our knowledge, this is the first time that PNC evolution is investigated in the context of brushite formation. The initial concentrations and lower pH conditions required for brushite formation may increase the fraction of bound Ca^2+^ as it is much higher than typically observed in hydroxyapatite formation.^11^

## Conclusions

**Figure 8:**
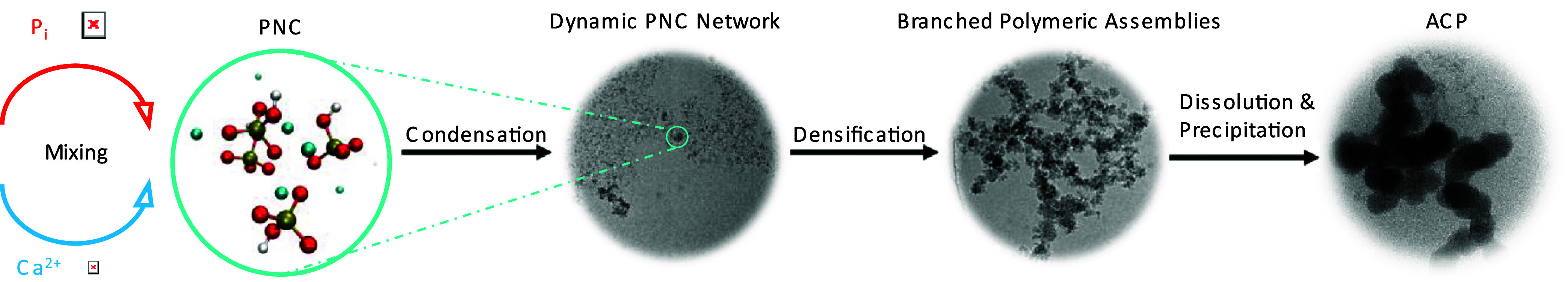
Scheme summarizing CaP mineralization process. Small PNC are formed initially followed by their aggregation into branched polymeric assemblies. Then, a dissolution followed by a local densification of spherical domains leads to ACP spheres.

Our study shows that CaP crystallization from aqueous solutions can be a remarkably complex process encompassing various transient species. The processes observed herein are summarized in Figure 8. Two precursors were identified corresponding to (*i*) small prenucleation clusters that initiate the mineralization by slowly evolving into (*ii*) “branched” polymeric assemblies by simple aggregation/dehydration. Interestingly, the latter species are “invisible” to ^31^P NMR, turbidimetry, or calcium-potentiometry. These transient entities have only scarcely been described,^8,13,46^ although we show that they can be stable for hours before suddenly undergoing a second phase transition. Our work further demonstrates that time-resolved SAXS, in combination with cryo-TEM, allows for a detailed characterization of these objects in terms of lifetime, size, and morphology. Their transition to solid particles arises from the dissolution of the clusters comprising the suprastructure and from local densification involving Ca^2+^ uptake into 100–200 nm circular/spherical domains. These domains densify until forming spherical ACP spheres of similar dimensions, which constitute the first nucleated solid. Finally, the ACP spheres transform quickly into brushite platelets under our conditions.

Next to this complex mechanistic pathway, the most striking result is yet the time-dependence of these processes. The succession of events is very reproducible and accessible to different types of experiments, hinting toward the very early stage CaP prenucleation precursors as trigger entities for the sequential mineralization event. Interestingly, we identified similar species also under much higher oversaturation in our previous work using dissolution dynamic nuclear polarization.^35,47^

Finally, our study demonstrates that ACP nucleation does not necessarily proceed through the aggregation of prenucleation clusters but rather from a dissolution and reassembly process, leading to local densification concomitant with increased Ca^2+^ uptake. Thus, the question of whether the local densification is proceeding through the formation of a dense liquid phase remains open.

## Experimental Section

### Sample Preparation

Phosphate (P_i_) samples were prepared by dissolving K_2_HPO_4_ (≥98%, Sigma-Aldrich) in a 20 mM HEPES (≥99.5%, Sigma-Aldrich) solution H_2_O/D_2_O (90/10). Following, a CaCl_2_ solution (98+%, Arcos Organics) was prepared analogously. Solutions were degassed, and the pH was adjusted to 7.0 using a NaOH solution (1 M). The solutions prepared under these conditions were used for SAXS and NMR measurements.

### Real-Time NMR

^31^P NMR spectra were acquired at a resonance frequency of 202.5 MHz with a spectral width of 24.7 ppm, using 90° flip angles (12 ms) and averaging 16 FIDs over a time of 11 h. The lock solvent used for all experiments was D_2_O. Prior to Fourier transformation, all data were zero-filled to twice the original FID size and apodized using a Gaussian window function. Subsequent to the Fourier transformation, all data were baseline-corrected using a Piecewise Cubic Hermite Interpolating Polynomial. The NMR signals were then fitted to two Lorentzian functions using home-written scripts embedded in the MATLAB software package using the “fitNlorentzian.m” function to extract signal intensities and chemical shifts.

To obtain inflection points *x*50 and sigmoidality parameters σ of the chemical shift time traces, the data were fitted to the following function:
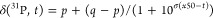
1

### p

with and *q* being fitting parameters.

The real-time *R*_2_ measurements were carried out in one measurement, acquiring *R*_2_ (gradient-selective spin–echo and CPMG) constants with three delays τ per *R*_2_ evaluation (τ = 0, 0.2, and 2 s), averaging 16 FIDs at a recovery delay of 5 s for each data point. For the CPMG experiment, a 180° pulse frequency of 50 Hz was employed to suppress influences of chemical exchange between different phosphate sites. To obtain *R*_2_, the resulting signal intensities *S*(τ) were then fitted to a monoexponential decay function.
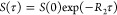
2

### Turbidimetry

Turbidimetry measurements were performed with a home-built turbidimeter using a SEN0189 turbidity sensor and a home-written Arduino control system for data readout. The experiments were performed at room temperature, employing a sampling rate of 1 s^–1^.

### Small-Angle X-ray Measurements (SAXS)

SAXS measurements were performed by prior mixing a 16 mM Pi solution with one of the prepared CaCl_2_ solutions and subsequently transferring and sealing the mixture into a glass capillary with 1.5 to 2 mm diameter and 10 μm wall thickness (Hilgenberg, Germany). X-ray patterns were measured using a microfocus X-ray source with a copper target (λ = 0.1542 nm) equipped with a pinhole camera (Nanostar, Bruker AXS) and a 2D position-sensitive detector (Vantec 2000). All two-dimensional SAXS patterns were radially averaged and background corrected to obtain the scattering intensities in dependence of the scattering vector q = 4π/l sin(θ), with 2θ being the scattering angle.

### Dynamic Light Scattering (DLS)

An in-house-built setup was used for the DLS measurements. The mixed solutions were placed in a focused laser beam (λ = 532 nm). A single-photon-avalanche-detecting MPD PDM-050-C0C connected to a buffered counter, implemented with an NI PCI-6601, was used to record consecutive time series of the scattered photons at a 90° angle. Measurements were performed at room temperature, using a channel width of 20 μs and a series length of 33 s.

The intensity is the sum of recorded events within one series. For each series, the normalized second-order autocorrelation was calculated and fitted with a modified Kohlrausch–Williams–Watts-function (KWW)  with  the normalized correlation function, B the baseline, τ the decay time, α the KWW parameter and β the coherence factor.

### Calcium Ion Potentiometry

Calcium ion concentration was monitored using a calcium-selective electrode (Ca-ISE, Metrohm No. 6.0508.110) connected to a Titrando titration device and analyzed with Tiamo 2.3 software (Metrohm). The Ca-ISE electrode was calibrated at room temperature using three solutions with respective calcium concentrations of 1, 5, and 15 mM, prepared in 20 mM HEPES buffer at pH 7.0. Before and between the measurements, the electrode was washed with acetic acid (0.1 M), distilled water, and recalibrated. In each reaction, a Ca-ion-containing solution (25 mL) was added to a Pi-containing solution (25 mL) with a 5 mL/min rate under slow stirring at 120 rpm. Data points per reaction were sampled at intervals of 1s.

### Cryo-TEM and EDS

Ca- and Pi-containing solutions were separately sterilized with a 0.22 mm syringe membrane filter (Sartorius) to remove any impurities that could interfere with the analysis of the cryo-TEM images. Similarly, as for potentiometry measurements, Ca^2+^-containing solution (5 mL) was added to Pi-solution (5 mL) under slow stirring at a rate of 120 rpm. Then, at specific time points, a drop (3 μL) of a reaction mixture was deposited on “quantifoil” (Quantifoil Micro Tools GmbH, Germany) carbon membrane grids, followed by absorbing excess liquid using filter paper, and finally quench-freezed in liquid ethane to form a thin vitreous ice film. The sample was transferred to the microscope operating at −180 °C. Cryo-TEM images were recorded on ultrascan 1000, 2k × 2k CCD camera (Gatan, USA), using a LaB_6_ JEOL JEM2100 (JEOL, Japan) cryo-microscope operating at 200 kV with a JEOL low-dose system (Minimum Dose System, MDS) to protect the thin ice film from any irradiation before and during the imaging.

X-ray energy-dispersive spectra (XEDS) were recorded using a JEOL (Japan) XEDS detector with 140 eV resolution using a JEOL 2100F (Japan) field-emission gun instrument operating at 200 kV under cryo-conditions.

### Powder X-ray Diffraction

Powder X-ray diffraction (PXRD) data were recorded on a Bruker D8 diffractometer equipped with a Lynx eye detector using a standard Cu tube (Ka = 1.54 Å) with measurement parameters including 40 kV, 20 mA, a 0.05° step size, and a range of 5–60° 2⊖ range.

## Supporting Information Available

Supplementary *d*-DNP, additional *in operandi* SAXS data, calcium potentiometry results of non-stirred solution, cryo-TEM images, EDX data, and X-ray diffractogram of final product (PDF)
