# Supplementary material for: Branched Polymeric Prenucleation Assemblies Initiate Calcium Phosphate Precipitation
Source: J Am Chem Soc. 2024 Sep 4;146(37):25614–24. doi: 10.1021/jacs.4c07325 (PMC11421018; doi:10.1021/jacs.4c07325)
Supplement: Supplementary file 1 — ja4c07325_si_001.pdf [file ja4c07325_si_001.pdf]

Supporting Information

Branched polymeric prenucleation assemblies initiate calcium phosphate precipitation

Ertan Turhan[1,2,#], Ieva Goldberga[3,#], Christopher Pötzl[1,2,#], Waldemar Keil[1,#], Jean-Michel Guigner[4], Martin F.T. Haßler[5,6], Herwig Peterlik[5], Thierry Azaïs[3] and Dennis Kurzbach[1,2]

[1]Institute of Biological Chemistry, Faculty of Chemistry, University of Vienna, Währinger Str. 38, 1090 Vienna, Austria

[2]University of Vienna, Vienna Doctoral School in Chemistry (DoSChem), Währinger Str. 42, 1090 Vienna, Austria

[3] Sorbonne Université, CNRS, Laboratoire de Chimie de la Matière Condensée de Paris (LCMCP), 4, Place Jussieu, F-75005 Paris, France

[4]Sorbonne Université, Institut de Minéralogie et Physique des Milieux Condensés (IMPMC), 4, Place Jussieu, F-75005 Paris, France

[5]Faculty of Physics, University of Vienna, Boltzmanngasse 5, 1090 Vienna, Austria

[6]University of Vienna, Vienna Doctoral School in Physics (VDS), Boltzmanngasse 5, 1090 Vienna, Austria

[#]equal contributions

Table of Contents

Figure SI1..1

Figure SI2..1

Figure SI3..2

Figure SI4..3

Figure SI5..4

Figure SI6..4

Figure SI7..5

Figure SI8..6

Figure SI9..7

Figure SI10..7

Figure SI11..8

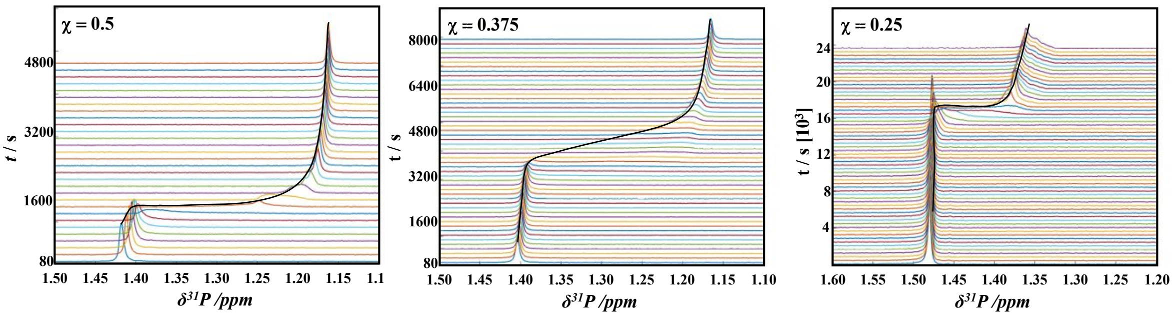

Figure SI1. Time-resolved ^{31}P NMR spectra for the three probed molar ratios.

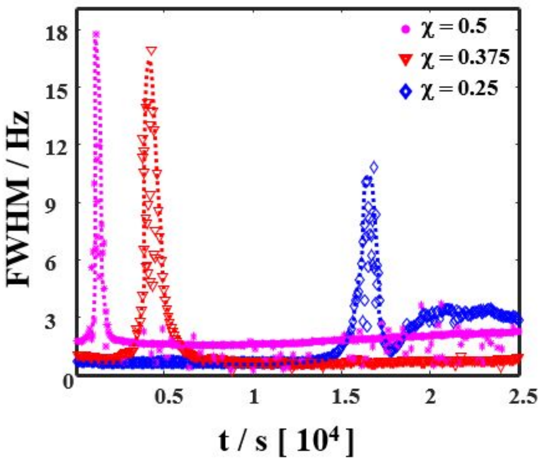

Figure SI2. Time-dependent linewidths (full width at half maximum) of the ^{31}P NMR spectra for the three probed molar ratios.

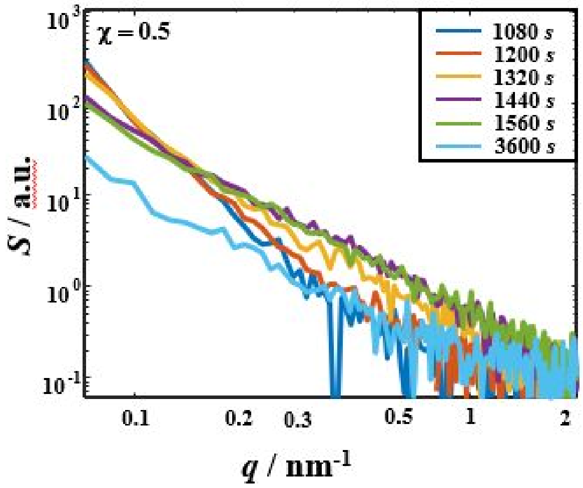

Figure SI3. Time-dependent SAXS scattering intensities for concentration χ = 0.5 in dependence on scattering vector q.

Fig. SI3 shows the measurement for the highest mole fraction χ = 0.5. 2D SAXS images were recorded for 120 s. Then, the 2D data were radially integrated and background corrected to result in SAXS intensities. Only a few curves were selected here to avoid the figure being too crowded and the information not being visible. Initially, up to 1320 s, intensities exhibit a strong decrease towards small *q*-values (slope -4 in the log-log diagram). This slope changes abruptly to -2 for the intensities recorded at 1440 s. Then, the data further decreases in intensity, but not in the slope, which remains constant at about -2. These data were fitted in the *q*-range 0.1 nm^{-1} < q < 1 nm^{-1}, using the power law function $I(q)=a\cdot q^{-n}$, with two parameters, amplitude *a* and fractal dimension *n,* as described in the main text.

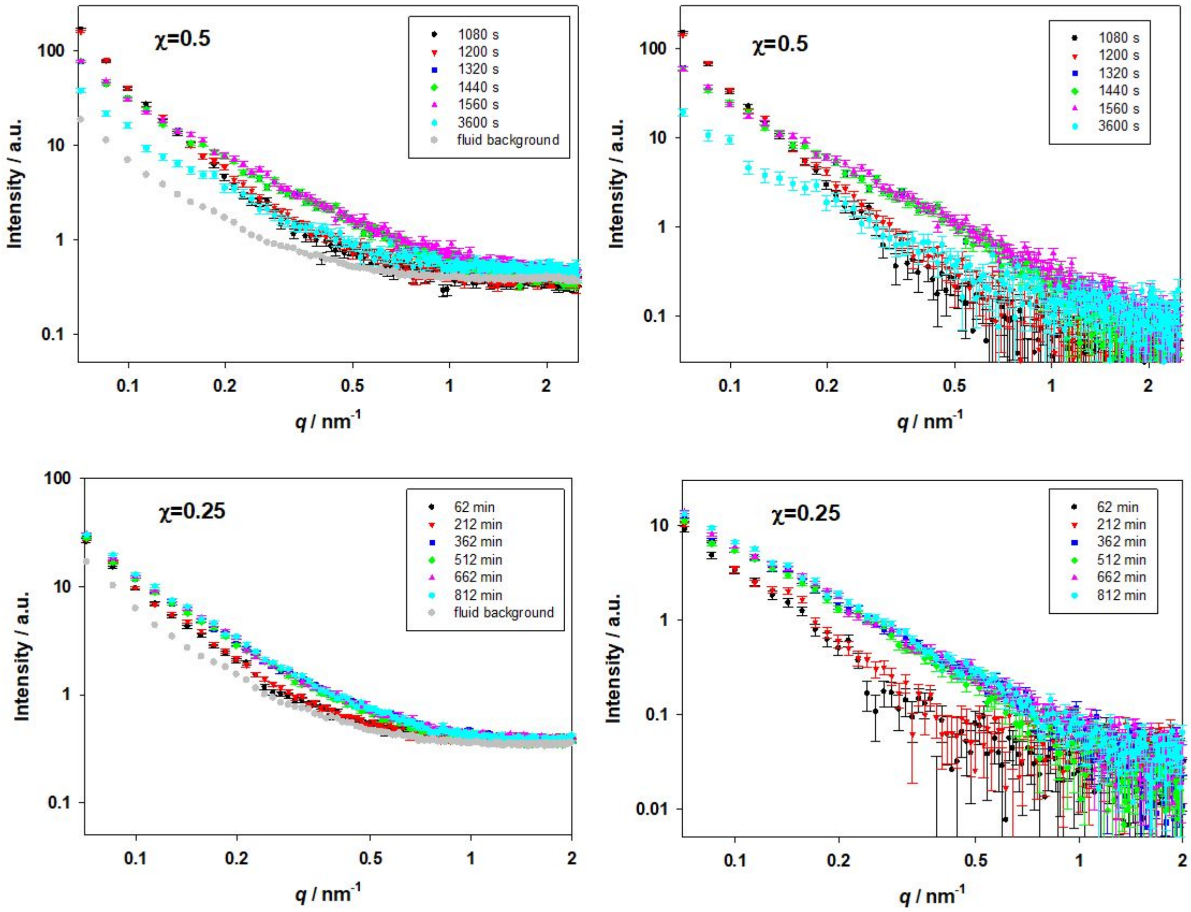

Figure SI4. Selected time-dependent SAXS scattering intensities (original data, left image, data after background subtraction, right image) for concentration χ = 0.5 (top) and 0.25 (bottom) in dependence on scattering vector q.

Fig. SI4 shows that the error of the fluid background can be neglected due to signal averaging for 1 hour and the resulting low background. In contrast, for the time-dependent measurement of the sample at χ = 0.5, an image was taken every two minutes. Yet, the intensity is still 10-fold higher than the background; this shows that the different power-law behavior of the scattering intensities reported in the manuscript is indeed a phenomenon related to the CaP system and not an instrumental artifact. Clearly, in Fig. SI3, the power law exponent changes from -4 to -2. After one hour, the total intensity decreases, but the power law exponent remains at -2. This is also clearly shown by the fit results of all data in Fig. 3 of the main text. The same power law behavior is also observed for the lowest concentration at χ = 0.25. However, the signal-to-background ratio diminishes from ca. ten to two, and the time between transitions increases considerably. Though this leads to an increasing fit error in Fig. 3 of the main text, in the integrated SAXS curves, the transition of the power law exponent from about 3.5-4 to 2 is still clearly evidenced.

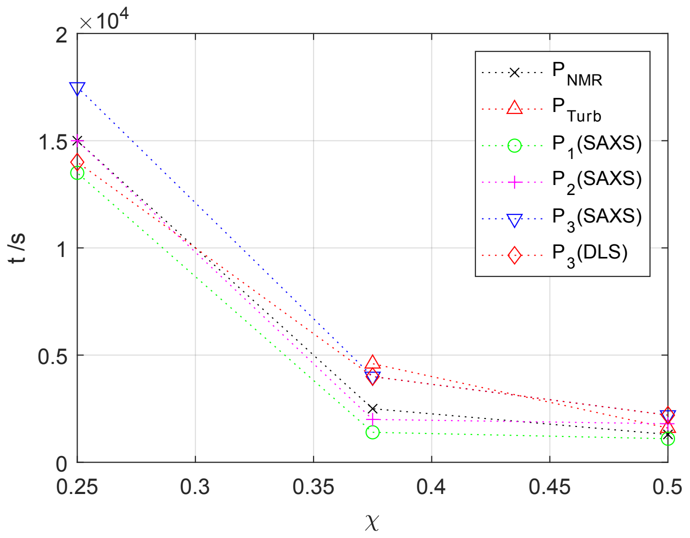

Figure SI5. Comparison of the time points P_3 observed for the three probed conditions by the different methods used.

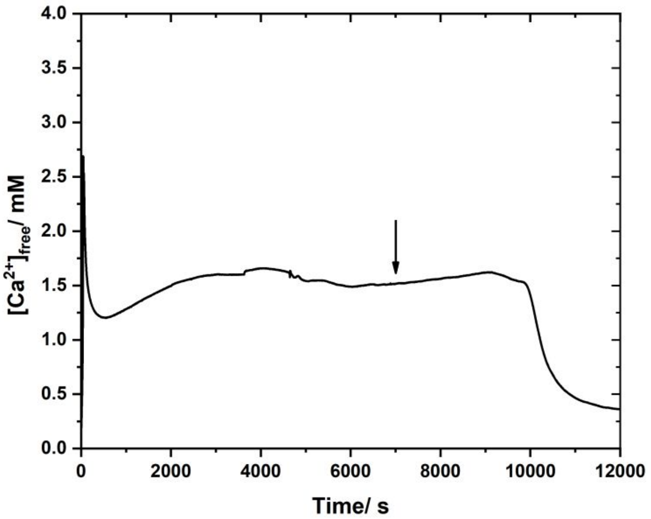

Figure SI6. Ca-potentiometry measurement without stirring after mixing P_i and Ca^{2+}-containing solutions (χ = 0.375). The arrow indicates the time when stirring was introduced (120 rpm).

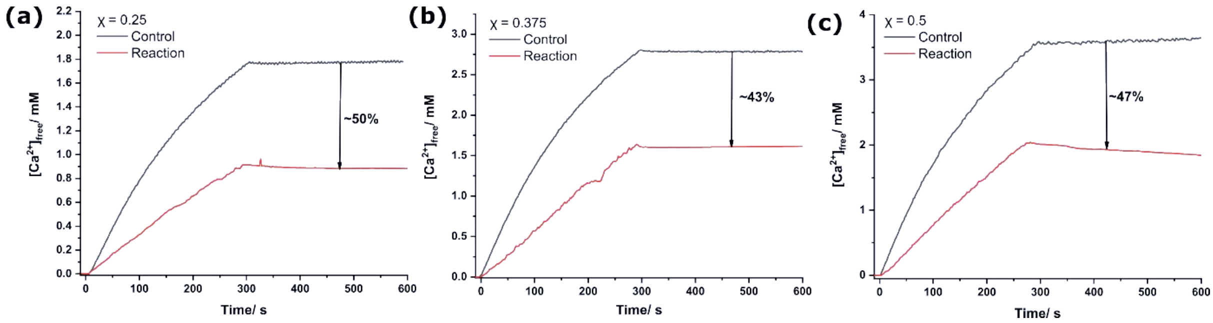

Figure SI7. Ca-potentiometry measurements comparing the mineralization experiments (red) and the control experiments without phosphate (black) to evaluate the influence of HEPES.

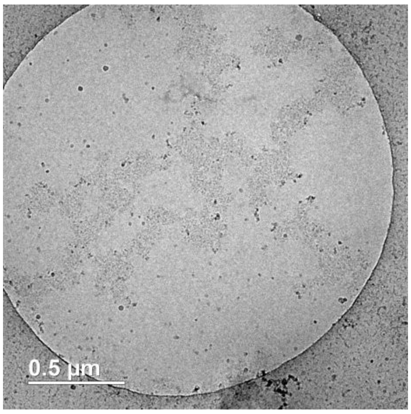

Figure SI8. Cryo-TEM observation depicting individualized PNC spread over micrometric domains (χ=0.5 after a few min of reaction).

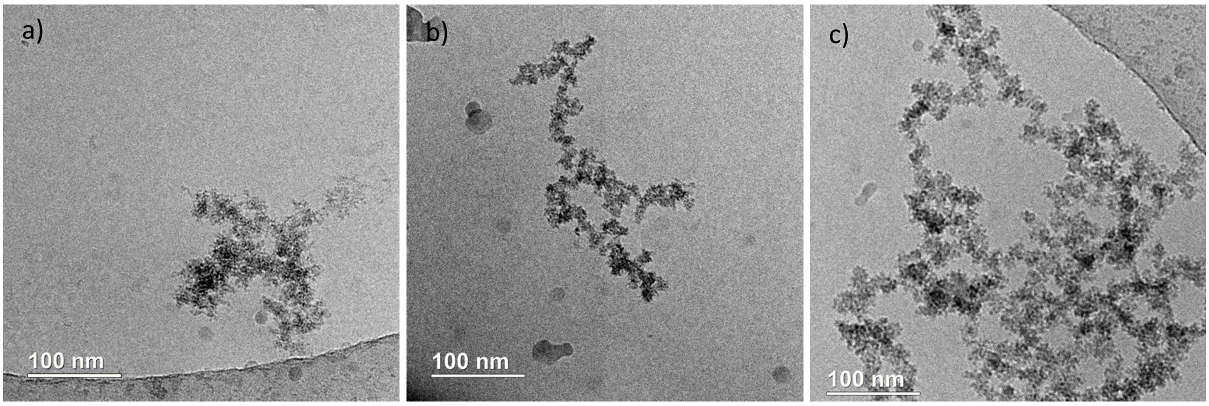

Figure SI9. Cryo-TEM observation of soluble branched polymeric aggregates for each condition: (a) χ = 0.25, (b) χ = 0.375 and (c) χ = 0.5.

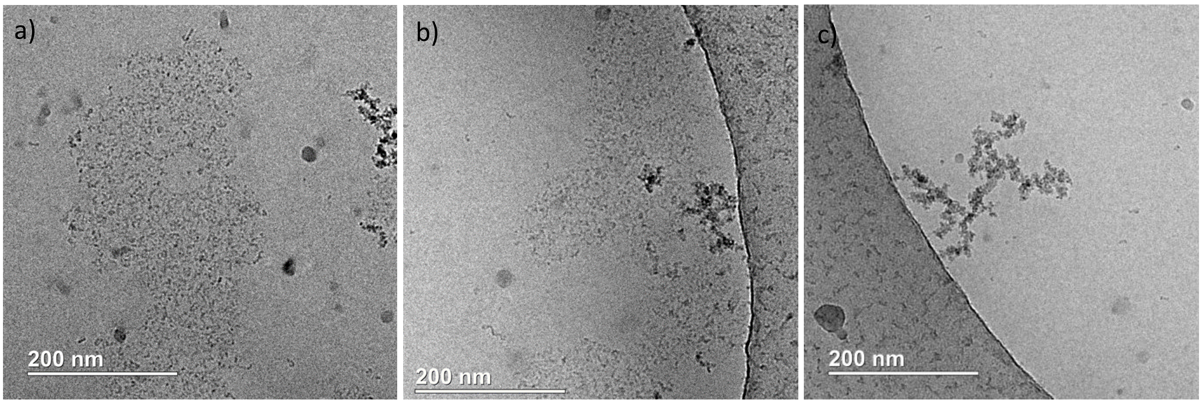

Figure SI10. Cryo-TEM observation of PNC and soluble branched polymeric aggregates in non-stirring conditions (χ = 0.375 after 4800 s of reaction).

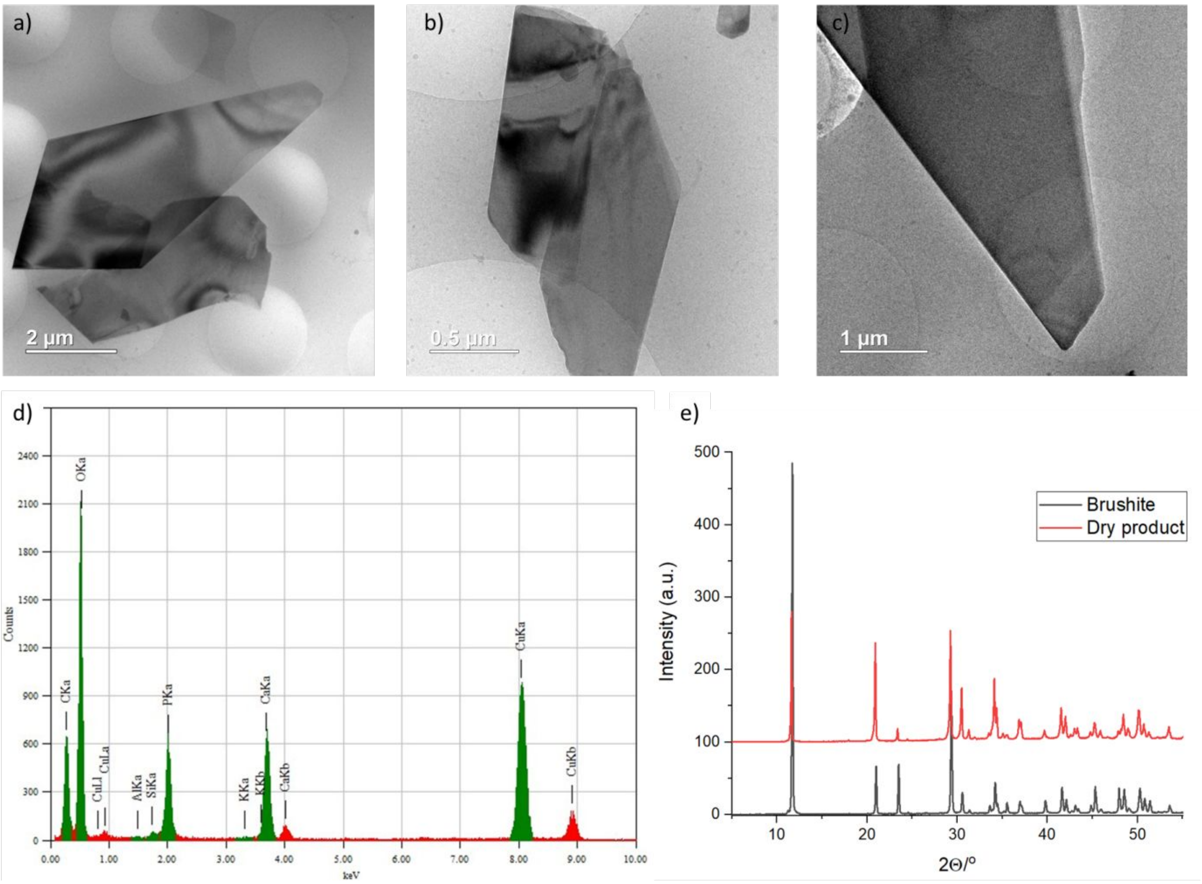

Figure SI11. Cryo-TEM observation of final mineralized products for the following conditions: (a) χ = 0.25, (b) χ = 0.375, and (c) χ = 0.5. (d) EDX analysis of the platelet particle indicating a Ca/P molar ratio of 1 (χ=0.375). (e) Powder XRD analysis of the final mineralized product shows brushite presence (χ=0.375).
